# Superficial Ventral Premotor Pathways to Primary Motor Cortex Shape the Temporal Coordination of Precision Grasping

**DOI:** 10.1111/ejn.70597

**Published:** 2026-07-02

**Authors:** Andrea Casarotto, Elisa Dolfini, Marta Russo, Giacomo Koch, Luciano Fadiga, Alessandro D'Ausilio

**Affiliations:** ^1^ IIT@UniFe Center for Translational Neurophysiology of Speech and Communication Istituto Italiano di Tecnologia Ferrara Italy; ^2^ Department of Neuroscience and Rehabilitation, Section of Physiology Università di Ferrara Ferrara Italy; ^3^ Institute of Cognitive Sciences and Technologies (ISTC) National Research Council (CNR) Rome Italy; ^4^ Experimental Neuropsychophysiology Lab Fondazione Santa Lucia IRCCS Rome Italy

**Keywords:** cortico‐cortical paired associative stimulation (cc‐PAS), Hebbian‐like plasticity, primary motor cortex (M1), reaching—grasping, ventral premotor cortex

## Abstract

Goal‐directed actions, such as picking up, manipulating, or using objects, are so ubiquitous that impairments in these skills can severely impact quality of life. Reaching‐grasping behaviors are driven by a frontoparietal network, with the ventral premotor cortex (PMv) and the primary motor cortex (M1) serving as critical frontal nodes. PMv‐M1 connectivity can be modulated using cortico‐cortical paired associative stimulation (cc‐PAS), which involves repeated paired transcranial magnetic stimulation (TMS) of both nodes. Stimulating M1 with an anterior–posterior (AP) current direction selectively enhances corticospinal excitability during isometric precision grip, but not during isometric power grip. However, it is unclear how the plasticity induction in the more superficial PMv‐M1 connectivity may influence the preparation and execution of goal‐directed, naturalistic reaching‐grasping actions. In this study, participants performed reaching‐grasping actions toward small or large objects, requiring precision or power grip, before and after applying the PMv‐M1 cc‐PAS_AP_ protocol. The plasticity‐induction protocol selectively modulated the joint angles temporal synergies during precision grip actions, suggesting a reorganization of whole‐arm reaching‐grasping coordination. The analyses of the joint angles spatial synergies did not reveal comparable effects. Taken together, these findings suggest that the PMv‐M1 plasticity‐induction protocol primarily modulated the temporal, rather than the spatial, control of joint angles recruitment during precision grip actions. Given that such basic skills are often permanently lost in stroke patients, our findings may offer valuable insight for the development of innovative therapeutic approaches for this clinical population.

## Graphical abstract

Goal‐directed reaching–grasping actions rely on PMv–M1 connectivity. Participants performed precision or power grips toward small or large objects before and after the PMv–M1 cc‐PAS_AP_ protocol. The PMv‐M1 plasticity induction produced selective changes in temporal joint‐angle synergies during precision grip, with no effect on spatial synergies, indicating that PMv–M1 plasticity primarily modulates temporal coordination of precise grasping actions.

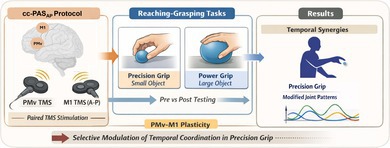

**Abbreviations**
AccP: acceleration peak
AccP/DecP: acceleration peak/deceleration peak ratio
AccT: acceleration time
AccT/DecT: acceleration time/deceleration time ratio
AP: anterior–posterior
AV: average velocity
cc‐PAS: cortico‐cortical paired associative stimulation
CNS: central nervous system
CSE: corticospinal excitability
DecP: deceleration peak
DecT: deceleration time
EMG: electromyography
FDI: first dorsal interosseus
M1: primary motor cortex
MEP: motor‐evoked potential
MGA: maximum grip aperture
MT: movement time
OSP: optimal scalp position
PA: posterior–anterior
PCA: principal component analyses
PMv: ventral premotor cortex
PV: peak velocity
RMS: root mean square
rMT: resting motor threshold
RT: reaction time
sp‐TMS: single‐pulse TMS
SSyn: spatial synergies
STDP: spike‐timing‐dependent plasticity
TMS: transcranial magnetic stimulation
TPV: time to the peak velocity
TSyn: temporal synergies

## 1 Introduction

Manipulating objects with the hands is one of the most common yet sophisticated actions performed by humans, requiring the temporal fine‐tuning of feedforward and feedback processes. The neural planning and control underlying these actions are supported by a cortical and subcortical network in which the ventral premotor cortex (PMv) and the primary motor cortex (M1) represent two critical nodes (Ehrsson et al. 2000; Murata et al. 1997; Prabhu et al. 2009; Raos et al. 2006). However, not all reaching and grasping actions are made equal and, in fact, different neuronal populations within M1 are specifically involved in the corticospinal control of fractionated finger control as opposed to whole‐hand power grasps (Muir and Lemon 1983). Precision grasping makes greater use of co‐contraction and of sensory feedback to improve endpoint accuracy (Gribble et al. 2003) and is selectively controlled via faster and direct corticospinal descending projections. Instead, whole‐hand control is also mediated by descending tracts characterized by greater divergence such as the reticulospinal projections (Tazoe and Perez 2017; Zaaimi et al. 2018).

Recently, it has been proposed that Transcranial Magnetic Stimulation (TMS) may be used to target the neural populations preferentially involved in the precision rather than in power grip, by changing the coil orientation (Casarotto, Dolfini, Fadiga, et al. 2023; Davis et al. 2022). In particular, cortico‐cortical paired associative stimulation (cc‐PAS), a TMS protocol that promotes Hebbian spike‐timing‐dependent plasticity (STDP) (Koch et al. 2013), in the PMv‐M1 network with an AP coil orientation over M1 (cc‐PAS_AP_) was shown to condition the neural populations preferentially recruited during isometric precision grip (Casarotto, Dolfini, Fadiga, et al. 2023).

However, conclusive evidence regarding the selective targeting of precision grip motor circuits is currently lacking. Such conclusions can be drawn by assessing the effect of neuromodulatory interventions on goal‐directed, naturalistic reaching‐grasping behaviors. In this context, detailed movement analysis may reveal *how* these actions are planned and executed, reflecting the fine balance between feedforward and feedback motor control processes.

In the present work, we recorded upper limb and hand kinematics of the participants while they performed precision and power grip actions before and 30 min after the administration of PMv‐M1 cc‐PAS_AP_. To study different action preparation processes (i.e., more general vs. specific), in half of the trials the participants were informed about the action (precision vs. power grip) to perform before the “Go” signal (“Informed” trials). In the other half, the action was specified only at the time of the “Go” (“Not‐Informed” trials; Jakobson and Goodale 1991). In addition to the traditional movement kinematic features, we observed the relationship between the acceleration and the deceleration phase in terms of magnitude and duration during the reaching phase. We hypothesized that PMv‐M1 cc‐PAS_AP_ would modulate this relationship reflecting a change in the temporal coordination of whole‐arm joint kinematics and consequently in *how* the neural control of movement is organized (e.g., shoulder, elbow, or wrist; Alexander and Crutcher 1990; Kakei et al. 1999). To explore this possibility, we analyzed whole‐arm joint angles profiles with a multidimensional decomposition technique (temporal and spatial principal component analysis—PCA; Berger et al. 2020; Russo et al. 2014; Santello et al. 1998). These data provide complementary evidence that noninvasive plasticity‐induction in the PMv‐M1 circuit may determine remarkable reorganization of the neural network involved in planning and controlling goal‐directed functional action units.

## 2 Methods

### 2.1 Ethical Approval

All the participants were informed about the experimental procedure and gave their written consent according to the *Declaration of Helsinki*. The experiment was approved by the ethical committee “Comitato Etico Unico della Provincia di Ferrara” (Approval No. 170592). The participants were compensated for their participation with €30.00.

### 2.2 Participants

A total of 19 healthy volunteers (mean ± SD age, 23.84 ± 1.14 years; 9 males; Table 1) took part in this study. The sample size was determined based on previous studies employing similar experimental paradigms and methodologies (Buch et al. 2010, 2011; Casarotto, Dolfini, Fadiga, et al. 2023, 2023; Davare et al. 2009; Koch et al. 2013). All the participants were included in the motor‐evoked potential (MEP) and 18 in the kinematic analyses. Due to technical problems during the kinematics recording, the extraction of the temporal and spatial synergies was conducted on the data of 13 participants. However, this sample size was deemed adequate based on previous studies (Berger et al. 2020; d'Avella et al. 2006, 2011).

**TABLE 1:** The rMT value indicates the percentage of the maximum stimulator output and is reported for all coils used. During the PMv‐M1 cc‐PAS_AP_ protocol the 50‐mm coil used for the rMT_AP_ was positioned on M1 while the 50‐mm coil used for the rMT_PA_ was on PMv.

Subjects (male)	Age	M1 rMT_AP_	M1 rMT_PA_
19 (9)	23.84 ± 1.14	50.37 ± 5.38	44.32 ± 4.77

### 2.3 Experimental Task

**FIGURE 1:**
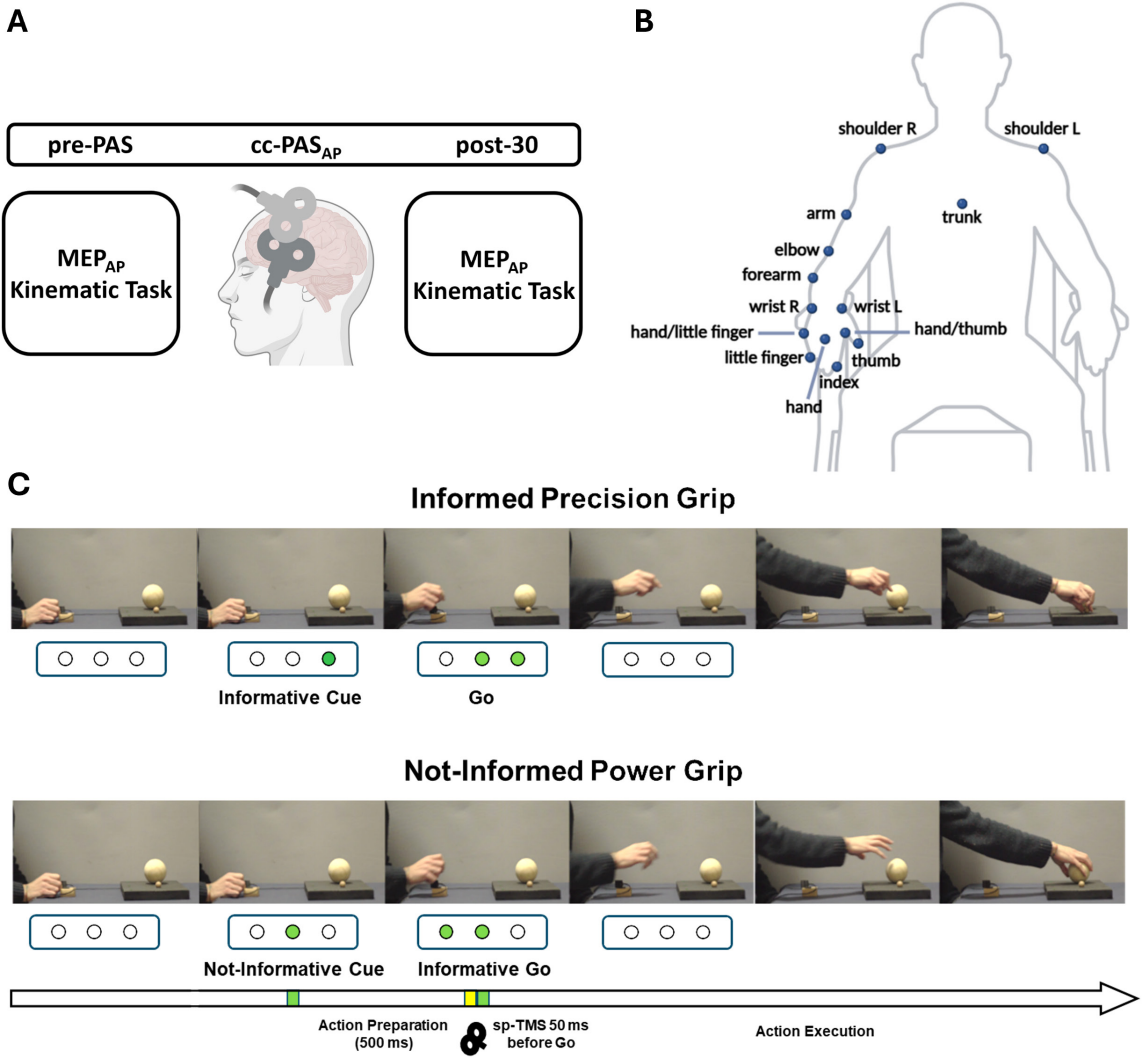
Behavioral task and experimental procedure. (A) Summary of the experimental procedures. The PMv‐M1 cortico‐cortical paired associative stimulation (PMv‐M1 cc‐PAS_AP_) was preceded by the pre‐PAS acquisition and followed by the post‐30 reacquisition. Each of these sessions involved the acquisition of MEPs in the preparation phase and the performance of the task shown below. (B) The panel shows the position of all markers used for recording kinematics during the task. (C) The panel shows an example of the experimental conditions. Snap shots of the two actions during the task are shown. The precision grip (*top*) and power grip (*bottom*) are aligned with the sequence of switching on of the LEDs in the case of the “Informed” (*top*) and “Not‐Informed” (*bottom*) conditions. This design involved a total of four conditions. *Yellow square* represents the single‐pulse TMS (sp‐TMS) timing used to evaluate corticospinal excitability (CSE) during the action preparation phase (i.e., 50 ms before the “Go” signal).

A small sphere (20‐mm diameter) that can be grasped with a precision grip and a large sphere (80‐mm diameter) that can be grasped with a power grip were positioned on a support in front of the subject at 30% of the subject's arm length from the right‐hand starting point. Three green LEDs were placed in front of the two spheres: one near each of them and one in between (Figure 1C). Participants started each trial holding a touch sensor between their thumb and index. They were instructed to perform a reach‐to‐grasp action based on the switching on of the LEDs. Once they grasped the sphere indicated by the corresponding LED, they had to lift it slightly, reposition it, and return to the starting position. Participants were asked to perform actions in a natural manner—not necessarily as fast as possible. This choice was made to investigate potential modulations in a naturalistic set, without experimental constraints.

In half of the trials, they were not informed about the action to perform (i.e., precision vs. power grip) until the “Go” signal (“Not‐Informed” trials). In this scenario (i.e., “Not‐Informed Precision Grip” and “Not‐Informed Power Grip”), the trial started with the central LED (“Not‐Informative Cue”) and after 500 ms one of the two LEDs near the spheres switched on (“Informative Go”).

In the other half of trials (“Informed” trials), the instruction was provided before the “Go” signal (“Informed” trial—i.e., “Informed Precision Grip” and “Informed Power Grip”). The trial started with the switching on of one of the two LEDs near one of the spheres (“Informative Cue”). After 500 ms, a “Go” signal (the central LED) prompted the participant to start the action. The delay between the first and the second LEDs was kept constant to allow robust preparatory activity, highly specific for informed trials and unspecific for uninformed trials.

A total of 160 trials were collected, 40 for each condition. In half of the trials of each experimental condition, 50 ms before the “Go”/“Informative Go” signal, a single‐pulse TMS (sp‐TMS) was administered to evaluate the corticospinal excitability (CSE) during the action preparation phase (Figure 1A–C).

### 2.4 Kinematic Data Recording

The reaching‐grasping actions were continuously recorded using a 10‐camera motion capture system (Vicon Nexus; RRID:SCR_015001; sampling rate: 100 Hz). The 3D position of 14 retro‐reflective markers was recorded. Eight markers with a diameter of 9.5 mm were used to record the trunk, upper arm, and wrist kinematics, while six markers with a diameter of 6.4 mm were used to record the hand and finger movements in the 3 axes (anteroposterior, X; mediolateral, Y; and vertical, Z).

Markers were placed at the following anatomical locations (Figure 1B): the sternum body (named “trunk”), the left acromial process (named “shoulder L”), the right acromial process (named “shoulder R”), the right triceps muscle (named “arm”), the lateral condyle of the humerus (named “elbow”), the right extensor digitorum muscle (named “forearm”), the styloid process of the radius (named “wrist L”), the ulnar epiphysis (named “wrist R”), the first dorsal interosseous (named “hand/thumb”), the condyle of V metacarpal bone (named “hand”), the condyle of III metacarpal bone (named “hand/little finger”), the last thumb phalanx (named “thumb”), the last index phalanx (named “index”), and the last little finger phalanx (named “little finger”).

### 2.5 Transcranial Magnetic Stimulation

Single‐pulse TMS and the PMv‐M1 cc‐PAS_AP_ protocols were administered through a 50 mm figure‐of‐eight focal coil connected to a Magstim BiStim2 monophasic stimulator (The Magstim Company, Whitland, UK). The first dorsal interosseus (FDI) optimal scalp position (OSP) was found by moving the coil in 0.5‐cm steps over the left primary motor cortex hand area and using a slightly suprathreshold stimulus. Resting motor threshold (rMT) was defined as the lowest intensity that evoked an MEP with > 50‐μV amplitude in five of 10 consecutive trials while the participants kept the FDI muscle relaxed (Rossi et al. 2009; Rossini et al. 2015). The individual OSP and rMT were defined for each coil used and marked on a cap. For each behavioral session (e.g., “pre‐PAS” and “post‐30”), during the action preparation phase we recorded 20 MEPs for each experimental condition (i.e., “Informed Precision Grip”, “Not‐Informed Precision Grip”, “Informed Power Grip”, “Not‐Informed Power Grip”).

### 2.6 Cortico‐Cortical Paired Associative Stimulation

In the PMv‐M1 cc‐PAS_AP_ protocol, dual‐sites TMS repeatedly activates the connection between the left PMv and left M1. One hundred couples of pulses were delivered at a frequency of 0.25 Hz for ∼6 min. The left PMv was stimulated at 90% of individual rMT, while the left M1 was stimulated at 120% of rMT. In each pair, the M1 stimulation followed the PMv stimulation by 6 ms. The coil over the left M1 was placed tangentially to the scalp on the FDI OSP, at ∼ 45° with respect to the midline and rotated 180° to induce an AP current flow. To estimate the position of the left PMv, the neuronavigation was conducted on an anatomical template using the SofTaxic Navigator System (E.M.S., Electro Medical System, Bologna, Italy). The skull landmarks (nasion, inion, and two preauricular points) and 23 points on the scalp were digitalized through a Polaris Vicra optical tracker (Northern Digital, Canada). To stimulate the left PMv, the coil was placed over the estimated scalp region corresponding to the Montreal Neurological Institute (MNI) coordinates *x* = −52.8, *y* = 11.6, *z* = 25.1 (Casarotto, Dolfini, Cardellicchio, et al. 2023).

### 2.7 EMG Recording

Surface EMG was recorded from the right FDI muscle through a wireless system (Zerowire EMG, Aurion, Italy) with a tendon–belly montage. EMG signals were digitized (sampling rate: 2000 Hz; low pass and high pass filters: 1st order; 6 dB/octave; bandwidth: 10–500 Hz) and acquired by a CED Power1401‐3A board (Cambridge Electronic Design, Cambridge, UK). The acquired data were stored for offline analysis using the Signal 3.09 software (Cambridge Electronic Design, Cambridge, UK).

## 3 Data Analysis

### 3.1 Corticospinal Excitability

At first, we excluded from the analysis all trials that presented a peak‐to‐peak MEP amplitude of ≤ 0.05 mV (Faro Viana et al. 2024; Rossini et al. 1994, 2015; Schilberg et al. 2018, 2021; Sollmann et al. 2017; Turrini, Bevacqua, et al. 2023). After this, for each trial, we computed the ratio between the MEP peak‐to‐peak amplitude and the root mean square (RMS) of the 100 ms pre‐TMS window. This was done to avoid any modulation of the CSE due to the premovement level of muscle contraction. Indeed, although participants were instructed to stay relaxed before the movement, the request to hold the touch sensor between the thumb and finger could cause a slight muscle contraction. After this, the MEPs were z‐scored (i.e., $z=\frac{x-\mu }{\sigma };$ where *x* represents the value to standardized, the μ is the mean of the dataset and σ represents the standard deviation of the dataset).

To investigate the effects induced by different PMv–M1 cc‐PAS_AP_ protocols on the CSE during the preparation of different actions we computed a 3 × 2 repeated measured ANOVA with “Action Condition” (informed precision grip vs. informed power grip vs. not‐informed grips) and “Time” (pre‐PAS vs. post‐30) as factors. The data from the not‐informed grip conditions were collapsed because, at the time of MEPs acquisition, participants had not yet received information about the upcoming action. Therefore, the two conditions were functionally indistinguishable prior to the “Informative Go” signal. Significant interactions were further explored via Bonferroni‐corrected post hoc tests (*p* < 0.05).

### 3.2 Kinematics

To characterize the reaching‐grasping actions, we paid particular attention to the acceleration profile. We extracted the “acceleration peak” (AccP) and the “deceleration peak” (DecP) absolute magnitude as well as the “acceleration time” (AccT), computed as the time between the movement onset and the acceleration zero‐crossing, and the “deceleration time” (DecT), computed as the time between the zero‐crossing and the offset of the movement. To evaluate the asymmetry in the acceleration profile (Cooke et al. 1989; Teasdale and Schmidt 1991), we computed the ratio between the AccT and the DecT (AccT/DecT) and the ratio between the AccP and the DecP (AccP/DecP). These analyses allowed us to evaluate a possible imbalance in both the duration and magnitude of these two components.

In addition to the aforementioned analyses, we consider different “classic” features that are typically employed to explore the transport and the hand preshaping phases. Specifically, we analyzed the end‐point “peak velocity” (PV) and the “average velocity” (AV) during the reaching phase. The “reaction time” (RT), computed as the time elapsed between the “Go” Signal and the time in which end‐point velocity exceeded the 3% of PV. The “movement time” (i.e., the time needed to complete the actions—MT) was computed from the start (3% of PV) to the contact with the object. The “maximum grip aperture” (MGA) as the maximum aperture between the thumb and the index during the reaching phase, and the “time to the peak velocity” (TPV), expressed as a percentage of MT. All these variables (except the MGA) were extracted from the trajectory of the “wrist L” marker.

Here, for the sake of clarity, the *Analyses* and the *Results* sections will present only the findings of the analysis on the AccT/DecT ratio and on the AccP/DecP ratio. These variables may permit a synthesis of potential modulation in the acceleration and deceleration components of the movement. The complete results of the analysis on other movement features are available in the Supporting Information.

Kinematic trajectories were low‐pass filtered using a digital two‐order Butterworth filter with a cutoff frequency of 5 Hz. For the trial segmentation, the onset of movement was defined as the point at which participants reached 3% of peak velocity. The trials were then time normalized.

For each feature, we computed a 2 × 2 × 2 repeated measured ANOVA with the within‐subjects factors “Action” (precision vs. power grip), “Information” (informed vs. noninformed trial), and “Time” (pre‐PAS vs. post‐30). Significant interactions were further analyzed via Bonferroni‐corrected post hoc tests. The analysis will emphasize the main effect of “Time” and its interactions to explore the PMv‐M1 cc‐PAS_AP_‐induced modulations.

### 3.3 Spatial and Temporal Synergies Extraction

We compute the following joint angles profiles during the reach to grasp movements: shoulder abduction, shoulder flexion, shoulder rotation, elbow flexion, wrist deviation, wrist elevation, hand pronosupination, grip aperture (i.e., the angle formed between the thumb and the index) and the little finger flexion (i.e., the angle formed by the flexion of the little finger against the palm of the hand). All these joint angles profiles were computed through the Vicon ProCalc 1.3.0 software (Vicon Motion Systems Ltd. UK).

Joint angles data were segmented and aligned to the onset of the movement, digitally low‐pass filtered (10‐Hz cutoff), averaged and temporally normalized. We applied a principal component analysis (PCA) for the temporal synergies (TSyn) and spatial synergies (SSyn—extraction via SynergyAnalyzer, a MATLAB toolbox implemented to identify kinematic and muscular synergies (Russo et al. 2024)). We extracted sets with a number of TSyn and SSyn ranging from 1 to 9. More precisely, the first set included the first principal component (i.e., one synergy), the second set included the first two components (i.e., two synergies), and so on, up to nine components. We then quantify the quality of data reconstruction with a *R* ^*2*^ value defined as:

$$R^{2}=1-\frac{\mathrm{SSE}}{\mathrm{SST}}=1-\frac{\sum_{k,t}{\left\|j\left(t,\vartheta_{k}\right)-\hat{j}\left(t,\vartheta_{k}\right)\right\|}^{2}}{\sum_{k,t}{\left\|j\left(t,\vartheta_{k}\right)-\bar{j}\right\|}^{2}}$$

where the SSE is the sum of the squared residual of the joint angles profiles estimated as a combination of joint angle synergies [$\hat{j}\left(t,\vartheta_{k}\right)$] with respect to the joint angles profiles observed $\left[j\left(t,\vartheta_{k}\right)\right]$ at time *t* for movement direction $\theta_{k}$. The SST is the sum of the squared residual of the joint angle profiles with respect to the mean profile. Thus, *R* ^2^ represents the fraction of total variation accounted by the synergy reconstruction. The first two synergies of each subject were selected, as these were found to explain at least 90% of the variance of the data in each subject.

Our objective was to investigate whether the temporal and spatial structures in the multijoint patterns of post‐30 actions differ from those observed in pre‐PAS data acquisition. If no differences exist, we expect that the temporal structures derived from each participant's pre‐PAS data could reconstruct both the pre‐PAS and post‐30 data effectively. Therefore, the TSyn and SSyn extracted from the pre‐PAS data of each subject were used to reconstruct the pre‐PAS and post‐30 session power and precision grip data of every subject separately (Berger et al. 2020; Figures 3C and 4C). Changes in reconstruction accuracy, above and beyond that expected due to intersubjective variability, are taken as evidence that plasticity induction protocols altered the temporal coordination pattern.

To maximize the quality and reliability of the TSyn and SSyn extraction through PCA, data from the “Informed” and “Not‐Informed” conditions were aggregated. This procedure increased the total number of available observations for each of the two action types (precision vs. power grip), thereby enhancing the statistical stability of the extracted principal components (Federolf 2016; Jolliffe 2002; Troje 2002).

We computed a 2 × 2 repeated measured ANOVA on the average *R* ^*2*^ of each subject with “Action Condition” (precision vs. power grip) and “Time” (pre‐PAS vs. post‐30) as within‐subjects factors. Significant interactions were further analyzed via Bonferroni‐corrected post hoc tests.

## 4 Results

### 4.1 Corticospinal Excitability

**FIGURE 2:**
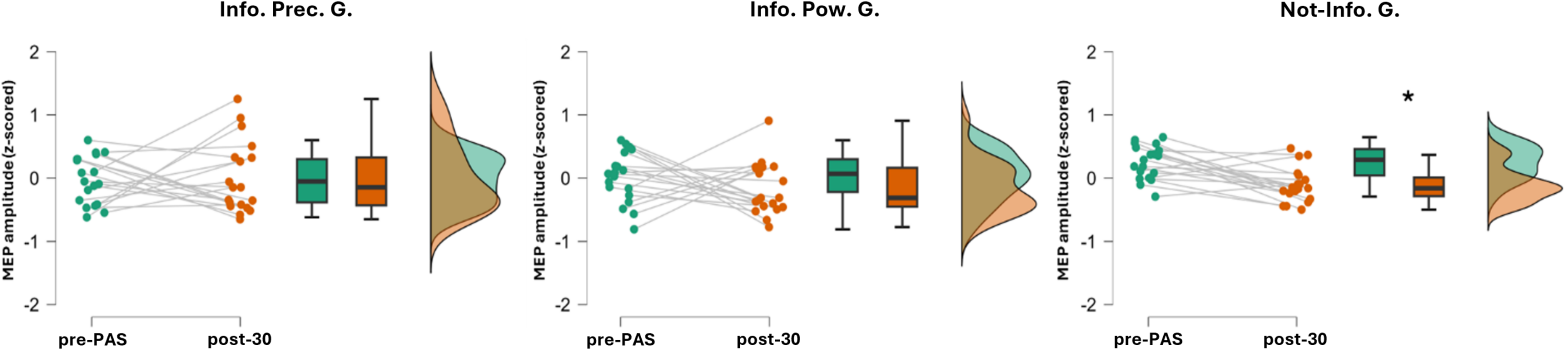
Effect of cc‐PAS_AP_ on the MEP acquired during the action preparation. Corticospinal excitability is expressed as the ratio between the MEP amplitude and the RMS calculated in the 100 ms before the TMS pulse. Info. Prec. G., Informed Precision Grip; Info. Pow. G., Informed Power Grip; Not‐Info. G., Not‐Informed Grip. The ratio was then z‐scored. Error bars represent the 95% CI; *p < 0.05.

The 3 × 2 ANOVA on MEP amplitude showed no significant main effect of “Action Conditions” (*F*_2,36_ = 1.88; *p* = 0.17; $\eta_{p}^{2}$ = 0.09) or “Time” (*F*_1,18_ = 2.03; p = 0.17; $\eta_{p}^{2}$ = 0.10). However, the interaction between these two factors was significant (*F*_2,36_ = 3.71; *p* = 0.03; $\eta_{p}^{2}$ = 0.17). The Bonferroni‐corrected post hoc analyses showed a significant reduction of the MEPs during the preparation of the “Not‐Informed Grip” after the cc‐PAS_AP_ as opposed to the same condition before cc‐PAS_AP_ (pre‐PAS, M = 0.25, SD = 0.27; post‐30, M = −0.11, SD = 0.28; *t*_18_ = 3.93; *p* = 0.01; Figure 2).

### 4.2 Acceleration Profile

The ANOVA on the ratio between AccP and DecP showed no significant main effect of “Action” (*F*_1,17_ < 0.01; *p* = 0.98; $\eta_{p}^{2}$ < 0.01), “Time” (*F*_1,17_ = 0.06; *p* = 0.80; $\eta_{p}^{2}$ < 0.01), or “Information” (*F*_1,17_ < 0.01; *p* = 0.96 $\eta_{p}^{2}$ = 0.01). At the same time, no significant interaction emerged between “Information” and “Action” (*F*_1,17_ = 2.46; *p* = 0.14; $\eta_{p}^{2}$ = 0.13), “Information” and “Time” (*F*_1,17_ = 1.22; *p* = 0.29; $\eta_{p}^{2}$ = 0.07), as well as “Action” and “Time” (*F*_1,17_ = 1.21; p = 0.29; $\eta_{p}^{2}$ = 0.07). The interaction between the three factors emerged as significant (*F*_1,17_ = 6.41; *p* = 0.02; $\eta_{p}^{2}$ = 0.27) but the Bonferroni‐corrected post hoc revealed nonsignificant modulations of the AccP/DecP ratio between the pre‐PAS and the post‐30 session.

The analyses on the AccT/DecT ratio showed a significant main effect of “Information” (“Informed”, M = 0.95, SD = 0.20; “Not‐informed”, M = 0.92, SD = 0.19; *F*_1,17_ = 14.44; *p* = 0.001; $\eta_{p}^{2}$ = 0.46) with higher AccT/DecT ratio observed in informed trials, indicating a longer acceleration phase in these trials. No significant main effect of “Action” (*F*_1,17_ = 1.39; *p* = 0.25; $\eta_{p}^{2}$ = 0.08) and “Time” (*F*_1,17_ = 2.05; *p* = 0.17; $\eta_{p}^{2}$ = 0.11). Moreover, no significant interaction emerged between “Information” and “Action” (*F*_1,17_ = 0.23; *p* = 0.64; $\eta_{p}^{2}$ = 0.01), “Information” and “Time” (*F*_1,17_ = 0.29; *p* = 0.60; $\eta_{p}^{2}$ = 0.02), “Action” and “Time” (*F*_1,17_ = 1.68; *p* = 0.21; $\eta_{p}^{2}$ = 0.09), or between the three factors (*F*_1,17_ = 0.47; *p* = 0.50; $\eta_{p}^{2}$ = 0.03).

### 4.3 Temporal Synergies

**FIGURE 3:**
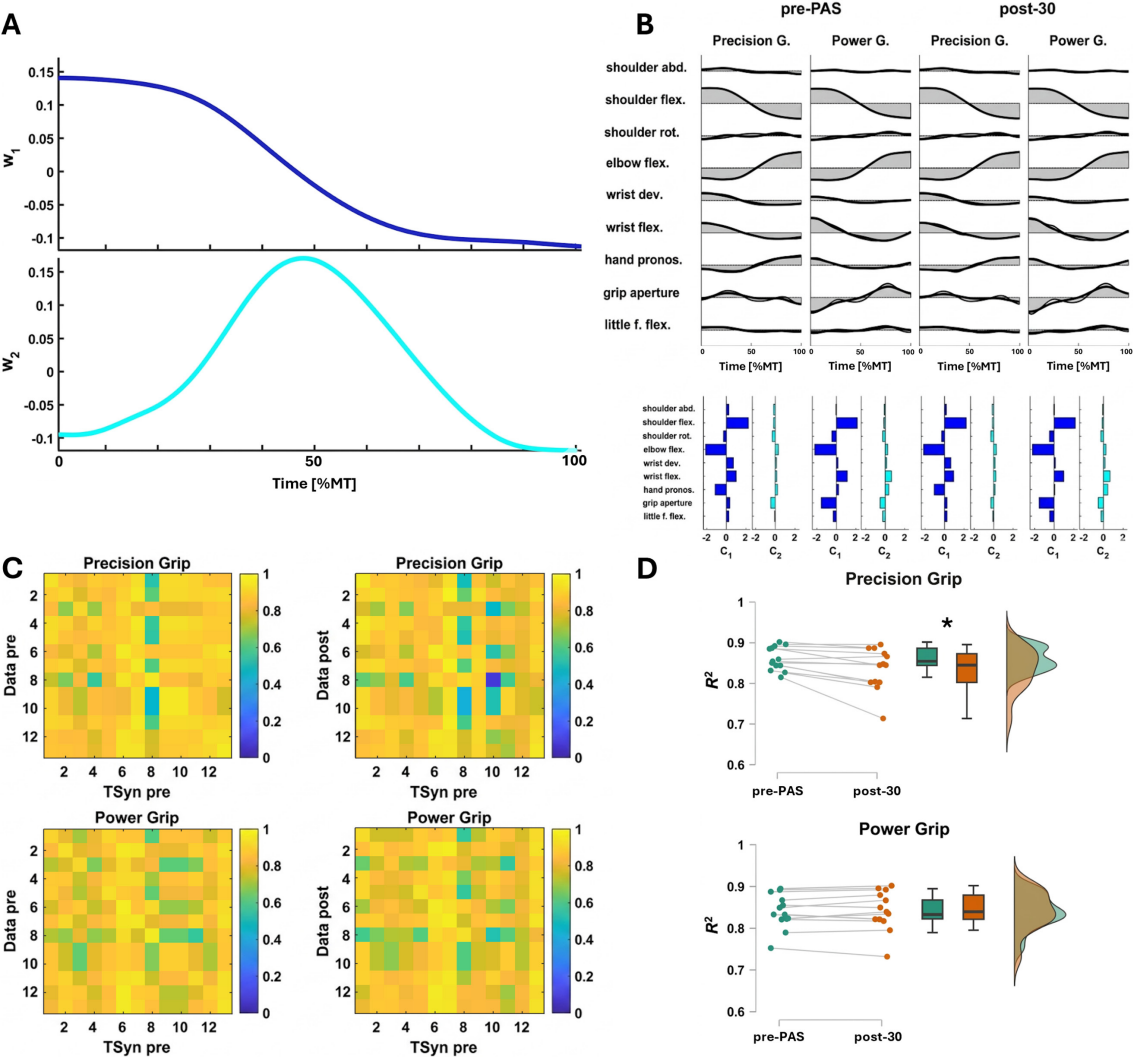
Joint angles profiles reconstruction using temporal synergies (TSyn). (A) The panel shows the first (W_1_—*blue*) and second (W_2_—*light blue*) TSyn of one representative subject. (B) Joint angles profiles of one subject reconstructed using a set of two TSyn (panel A) extracted from the same subject. In the *upper part* of the figure, the shaded area represents the original data; the thick line shows the reconstructed data. The *bottom part* of the figure shows the coefficients of each joint angle for the two TSyn across conditions. For each condition, joint angles coefficients of the first synergy (C_1_) are shown in *blue*, whereas those corresponding to the second synergy (C_2_) are shown in *light blue*. (C) Each panel shows the *R* ^*2*^ values obtained from all possible combinations of reconstructed joint angles data and TSyn. Rows correspond to individual subject data to be reconstructed, while column correspond to the set of TSyn derived from each subject and used for the reconstruction. The top panels show the reconstruction of the pre‐PAS (*right*) and post‐30 (*left*) data of precision grip action using the pre‐PAS temporal synergies. Similarly, the bottom panels show the reconstruction of the pre‐PAS (*right*) and post‐30 (*left*) data of the power grip action. (D) The box plots show the selective reduction of the quality of the post‐30 precision grip data reconstruction using the pre‐PAS TSyn. This was not the case for the power grip data, which showed comparable reconstruction quality to the pre‐PAS data. Error bars represent the 95% CI; *p < 0.05. Elbow flex., elbow flexion; Grip aperture., grip aperture; Hand pronos., hand pronosupination; Little f. flex., little finger flexion; Shoulder abd, shoulder abduction; Shoulder flex., shoulder flexion; Shoulder rot., shoulder rotation; Wrist dev., wrist deviation; Wrist flex., wrist flexion.

The Figure 3 shows illustrative profiles of the joint angles for one participant and the relative reconstruction obtained with two TSyns and the coefficients.

To evaluate differences in the temporal structures of multijoint patterns between the pre‐PAS and post‐30 sessions across the entire population, we computed the *R* ^*2*^ value for the reconstruction of each participant's data using synergies derived from the pre‐PAS session of all participants. We then compared the *R* ^*2*^ values of pre‐PAS reconstructions with those of post‐30 reconstructions, analyzing precision and power grips separately. As mentioned in the *Data Analyses* section, the “Informed” and “Not‐Informed” conditions were aggregated to increase the reliability of TSyn extraction and to focus the analysis specifically on the two distinct actions (precision vs. power grip). Figure 3C illustrates the *R* ^*2*^ values for each combination of participants and experimental conditions, while Figure 3D presents the average *R* ^*2*^ values for pre‐PAS and post‐30 reconstructions for both grip types. The 2 × 2 repeated measures ANOVA on the average *R* ^*2*^ showed a significant main effect of “Time” (*F*_1,12_ = 5.52; *p* = 0.04; $\eta_{p}^{2}$ = 0.32) but a nonsignificant main effect of “Action” (*F*_1,12_ = 4.44; *p* = 0.06; $\eta_{p}^{2}$ = 0.27). A significant interaction between “Action” and “Time” emerged (*F*_1,12_ = 25.72; *p* < 0.01; $\eta_{p}^{2}$ = 0.68). The Bonferroni‐corrected post hoc analysis showed a significant, and selective, reduction in the capacity (i.e., average *R* ^*2*^) of precision grip post‐30 data reconstruction, using the pre‐PAS TSyn (pre‐PAS, M = 0.86, SD = 0.03; post‐30, M = 0.84, SD = 0.05; *t*_12_ = 4.13; *p* = 0.01; Figure 3D). With the same set of TSyn, no significant difference was present in the capacity of the power grip data reconstruction between the pre‐PAS and the post‐30 (pre‐PAS, M = 0.84, SD = 0.04; post‐30, M = 0.84, SD = 0.05; t_12_ = −2.69; *p* = 0.12).

### 4.4 Spatial Synergies

**FIGURE 4:**
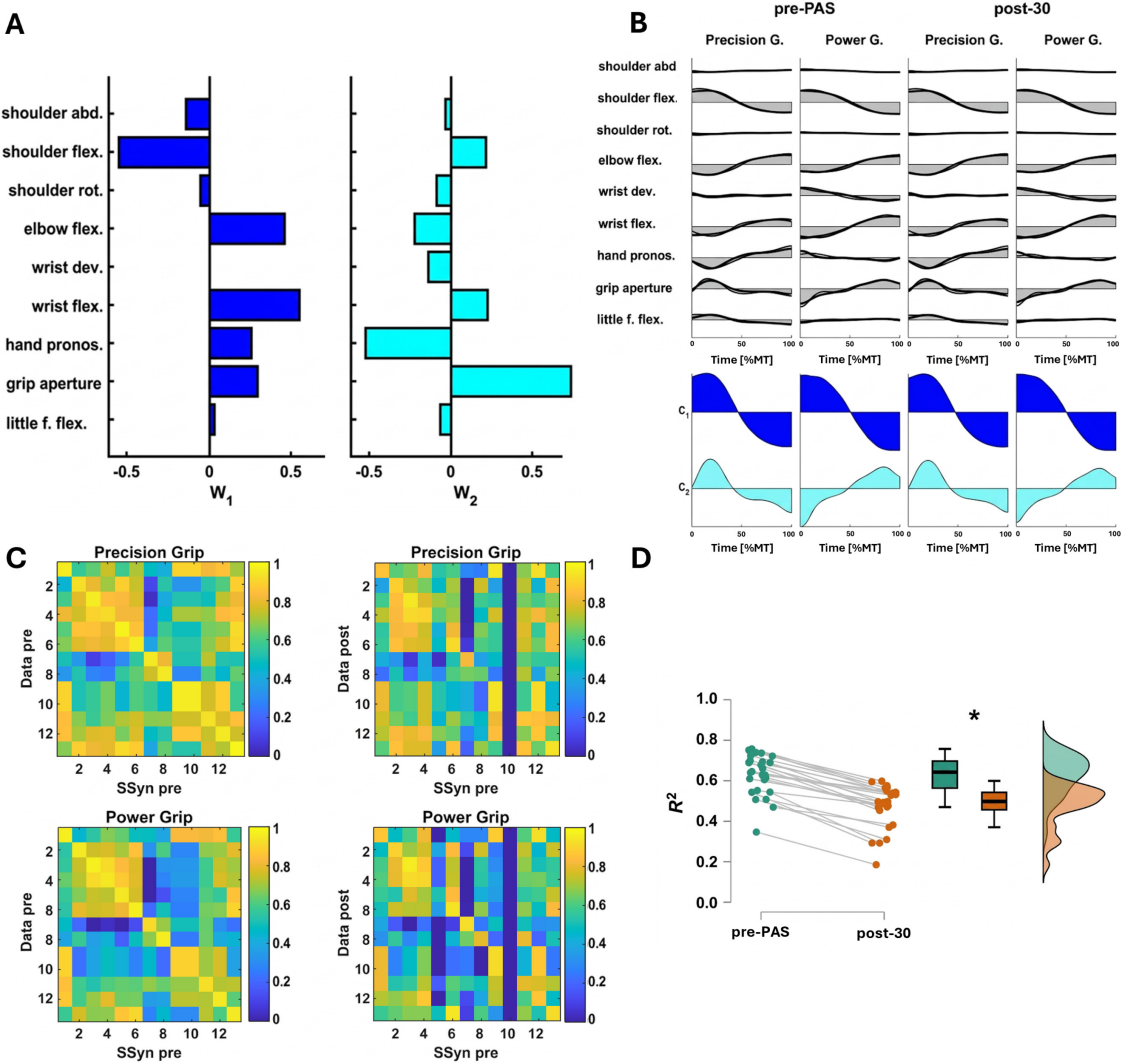
Joint angles profiles reconstruction using spatial synergies (SSyn). (A) The panel shows the first (W_1_—*blue*) and second (W_2_—*light blue*) SSyn of one representative subject. (B) Joint angles profiles of one subject reconstructed using a set of two spatial synergies (panel A) extracted from the same subject. The shaded area represents the original data; the thick line shows the reconstructed data, while the shaded area represents the original data. Below are shown the temporal coefficient of the first (C_1_—*blue*) and the second (C_2_—*light blue*) SSyn. (C) The panels show the *R* ^*2*^ values of all possible combinations of reconstructed joint angles data and synergies. The rows correspond to the data for each individual subject to be reconstructed, while the columns correspond to the set of SSyn values derived from each subject and used for the reconstruction. The *left* panels show the reconstruction of the pre‐PAS data using the pre‐PAS SSyn, while the *right* panels show the reconstruction of the post‐30 data using the pre‐PAS SSyn. (D) The box plot shows the reduction of the quality of the post‐30 data reconstruction using the pre‐PAS TSyn. Error bars represent the 95 CI; *p < 0.05. Grip aperture., grip aperture; Hand pronos., hand pronosupination; Little f. flex., little finger flexion; Elbow flex., elbow flexion; Shoulder abd, shoulder abduction; Shoulder flex., shoulder flexion; Shoulder rot., shoulder rotation; Wrist dev., wrist deviation; Wrist flex., wrist flexion.

The Figure 4C shows the *R* ^*2*^ values for each combination of participants and experimental conditions. The analyses on the average *R* ^*2*^ (Figure 4D) showed a significant main effect of “Time” (*F*_1,12_ = 24.17; *p* < 0.01; $\eta_{p}^{2}$ = 0.67; pre‐PAS, M = 0.63, SD = 0.09; post‐30, M = 0.47, SD = 0.10) and a significant main effect of “Action” (*F*_1,12_ = 177.72; *p* < 0.01; $\eta_{p}^{2}$ = 0.94). A nonsignificant interaction emerged between these factors (*F*_1,12_ = 0.61; *p* = 0.45; $\eta_{p}^{2}$ = 0.05).

## 5 Discussion

In this study, we gathered converging evidence demonstrating that PMv‐M1 cc‐PAS_AP_ induced a nontrivial reshaping of how precision grip actions are prepared and executed. Specifically, the PMv‐M1 plasticity‐induction protocol modulated the temporal control of joint recruitment, selectively during precision grip actions. These results are consistent with previous findings suggesting a preferential involvement of more superficial M1 neuronal populations—preferentially recruited by AP current direction—in the control of precision grip actions (Casarotto, Dolfini, Fadiga, et al. 2023).

Considering that these fine motor abilities are often irreversibly impaired in stroke patients, our findings provide new important insights for developing innovative therapeutic strategies for this clinical population.

### 5.1 Reshaping of Whole‐Arm Temporal Coordination

The PMv‐M1 cc‐PAS_AP_ induced a general shortening of RTs (Figure S3—Supporting Information), while the MGA was reduced in precision grip and increased in power grip “Informed” trials (Figure S4—Supporting Information). The latter being a particularly salient and well‐known feature differentiating the two types of grasps (Jeannerod 1984; Napier 1956; Smeets and Brenner 1999). In parallel, we observed also an action‐unspecific reduction in maximum acceleration and deceleration (Figure S1—Supporting Information) as well as a prolonged acceleration time (Figure S2—Supporting Information). These results can easily be interpreted as an effect of adaptation during the course of the experiment. Over repetitions, both a general reduction of RTs and a differentiation of the two motor plans, marked by the opposite modulation of MGA in the two actions, is to be expected in the “Informed” trials. The data on acceleration profiles support this possibility by showing greater smoothness of movement (i.e., reduced acceleration and deceleration peaks). The absence of action‐specific modulation of these macroscopic kinematic components can be readily explained considering the marked stereotypy of these actions when executed in a naturalistic manner. Therefore, it would not be realistic to expect a cc‐PAS‐induced modulation of these macroscopic features.

In contrast, other action‐specific modulations cannot be attributed to nonspecific short‐term adaptation processes. Instead, they may reflect the reshaping of the neural encoding of muscle and joint activation by the central nervous system (CNS) (Brown and Cooke 1990; Cooke and Brown 1994).

In this regard, the decomposition of joint angles activity into temporal and spatial synergies was employed. The concept of muscle and kinematic synergy has been proposed as a neural mechanism to simplify motor control by reducing the dimensionality of the motor commands (Bizzi et al. 2008; Bizzi and Cheung 2013; d'Avella et al. 2003; Lacquaniti et al. 2012; Mussa‐Ivaldi and Bizzi 2000; Tresch et al. 1999). Specifically, temporal synergies reflect coordination in time, and mapping of goals into high‐level features of motor commands (i.e., planning), whereas spatial synergies reflect the spinal organization to execute the movement (i.e., actuation; Brambilla et al. 2023).

These kinematic synergies, proposed as building blocks shared across different actions, have been analyzed using multidimensional decomposition techniques, such as PCA (Leo et al. 2016; Russo et al. 2014; Santello et al. 1998, 2013).

Based on these assumptions, motor impairments in cerebellar patients were successfully characterized by comparing their synergies with those of healthy subjects (Berger et al. 2020). Here, we used the same principle to describe the effects of plasticity induction in the PMv‐M1 network by comparing the joint angles SSyn and TSyn extracted after the PMv‐M1 cc‐PAS_AP_ with those expressed before the PMv‐M1 cc‐PAS_AP_ protocol.

The SSyn extracted from the pre‐PAS data showed a reduced capacity to reconstruct the post‐30 data across both actions. This likely reflects nonspecific effects of task repetitions, partially captured by discrete kinematic variables, and potentially related to a spinal level organization of motor actuation (Brambilla et al. 2023). In contrast, the TSyn extracted from the pre‐PAS data exhibited different reconstruction performance for the two actions. The pre‐PAS TSyn showed an equivalent capacity to reconstruct both the pre‐PAS and the post‐30 power grip data, whereas their capacity to reconstruct the post‐30 precision grip data was significantly reduced. This pattern indicates that PMv–M1 cc‐PAS_AP_ selectively modulates the cortically driven temporal organization of the precision reaching‐grasping functional unit (Brambilla et al. 2023). Given that motor synergies constitute the basic units shared across actions and serve to simplify motor control under continuously changing environmental demands, the observed dissociation between spatial and temporal synergies is particularly informative. The absence of grasp specificity for spatial synergies, together with the selective modulation of temporal synergies, suggests that the PMv–M1 interactions primarily contribute to the temporal orchestration of a largely stable spatial muscle–joint configuration. This result is consistent with the observation that activity in the motor and premotor areas strongly correlate with kinematic synergies (Leo et al. 2016). Moreover, our findings extend this framework by indicating a greater role of PMv–M1 circuitry in shaping the temporal coordinative structures underlying goal‐directed actions requiring fine precision control.

### 5.2 Action Preparation

Before the execution of a voluntary action, a series of sensory and motor processes interact with each other (Bestmann and Duque 2016) to form, select, and execute a motor plan that is congruent with the goal. As we argued before, the PMv‐M1 cc‐PAS_AP_ may determine the modulation of the precision grip action, in particular by promoting its late feedback‐based component.

Here we probed CSE at one key timing before the Go signal (Houdayer et al. 2012; Neige et al. 2021) and found no action‐specific modulation after the PMv‐M1 cc‐PAS_AP_ protocol, even if participants knew in advance which action to prepare. Nevertheless, before the PMv‐M1 plasticity induction, the CSE was tendentially larger when participants were not informed about the action to perform than when they were informed. This result might be explained by the competition of multiple concurrently active motor plans in these trials. It has been reported that, during the preparation phase, an increase in motor inhibition (Neige et al. 2021; or “impulse control”) is thought to prevent premature response initiation (Derosiere and Duque 2020; Duque et al. 2010; Labruna et al. 2014).

Here, maintaining two motor plans active at the same time might have attenuated the inhibition, resulting in a greater CSE. The application of the PMv‐M1 cc‐PAS_AP_ determined a significant reduction of the CSE only in the uninformed trials, as if the plasticity‐induction protocol could attenuate the impact of task demands (i.e., prepare two different actions) on inhibitory control. Indeed, PMv‐M1 cc‐PAS_AP_ modulates both fast and slow M1 GABAergic activity (i.e., GAGA_A_ and GABA_B_ receptors mediated; Casarotto, Dolfini, Cardellicchio, et al. 2023; Turrini, Fiori, et al. 2023), which is in agreement with monkey data showing that PMv projections specifically target M1 inhibitory interneurons (Ghosh and Porter 1988; Tokuno and Nambu 2000). Therefore, the release of GABAergic influence, driven by PMv‐M1 plasticity induction protocols may thus explain the effects we observe in trials requiring a greater deal of inhibitory control (i.e., uninformed trials).

## 6 Conclusions

This study extends our knowledge on how naturalistic grasping actions are organized. Specifically, by modulating the PMv‐M1 connectivity with cc‐PAS_AP_, we focused our attention on a key segment of a wider brain network engaged in these tasks. Previous research targeting the same anatomo‐functional network showed modulation of corticospinal motor output during the execution of isometric precision grip (Casarotto, Dolfini, Fadiga, et al. 2023) or measured the impact of stimulation on tasks testing general hand motor skills (Fiori et al. 2018). Crucially, we show for the first time that the PMv‐M1 plasticity induction modulates the whole‐arm temporal coordination of precision grip. These results are potentially relevant for clinical applications. In fact, this protocol could, for example, serve as a maintenance therapy to preserve fine motor skills in elderly populations at risk of progressive motor function decline or to augment traditional neuromotor rehabilitation in stroke patients (MacLennan et al. 2023; Martino Cinnera et al. 2025).

## 7 Limitations and Future Direction

The present study investigated the effects of PMv–M1 plasticity induction using a cc‐PAS protocol with an AP coil orientation over M1. This orientation preferentially recruits more superficial M1 neuronal populations (Aberra et al. 2020; Sommer et al. 2013), which are thought to be more engaged in fine precision control (Casarotto, Dolfini, Fadiga, et al. 2023). However, some limitations should be acknowledged. The absence of a comparison group in which plasticity was induced with a different coil orientation (e.g., posterior–anterior; PA) limits the possibility of comparing these results. Future studies should directly compare AP and PA coil orientations to determine whether distinct PMv–M1 pathways differentially shape movement kinematics. In addition, the present work focused on kinematic outcomes, without a direct assessment of muscle activity. Incorporating electromyographic recordings in future experiments would allow evaluation of whether different PMv–M1 cc‐PAS_AP_ protocols induce distinct modulations at the level of muscle activation.

## Author Contributions

**Andrea Casarotto:** conceptualization, data curation, formal analysis, investigation, methodology, project administration, software, visualization, writing – original draft, writing – review and editing. **Elisa Dolfini:** conceptualization, investigation, methodology, visualization. **Marta Russo:** methodology, software, writing – review and editing. **Giacomo Koch:** conceptualization, methodology, supervision, visualization, writing – review and editing. **Luciano Fadiga:** conceptualization, funding acquisition, supervision, writing – review and editing. **Alessandro D'Ausilio:** conceptualization, funding acquisition, methodology, project administration, supervision, visualization, writing – original draft, writing – review and editing.

## Funding

This work was supported by the Ministero dell'Università e della Ricerca (PRIN 2020: 20208RB4N9), the NextGenerationEU, https://doi.org/10.13039/100031478, MOTUS: P2022J8AXY, PRIN 2022: 2022XW3MJX, and the HORIZON EUROPE Digital, Industry and Space (HORIZON‐CL4‐2022‐DIGITALEMERGING‐02‐101120727).

## Conflicts of Interest

The authors declare no conflicts of interest.

## Supporting information




**Figure S1:** **Main effect of “Time” on the AccP and DecP.** The *left* panel shows the modulation in the AccP magnitude. The *right* panel shows the modulation of the DecP magnitude. After the PMv‐M1 cc‐PAS_AP_ we observe the reduction of the AccP and DecP magnitude. Error bars represent the 95% CI; *p < 0.05.
**Figure S2: “Time” main effect in the AccT.** The main effect of time on the AT. After the PMv‐M1 cc‐PAS_AP_ we observe an increase in the AccT. Error bars represent the 95% CI; *p < 0.05.
**Figure S3: Modulation of the RT between the pre‐ and post‐30 sessions.** Participants showed a significant reduction in RT in all conditions. As mentioned above, participants were instructed to perform a natural movement. This led to relatively long RTs but allows us to evaluate modulations without any experimental constraint. Error bars represent the 95% CI; *p < 0.05.
**Figure S4: Modulation of the MGA between pre‐and post‐30 session.** The *left* panel shows a significant reduction in of the MGA in the “Informed” precision grip. The *right* panel shows the opposite trend in the power grip. Indeed, after the PMv‐M1 cc‐PAS_AP_, there is a significant larger MGA. Here, the precision and power grip are plotted separately as the MGA strictly depend by the dimensions of the target. Collapsing the conditions into a single “Informed” condition would not be informative of the effects induced in the two actions. Error bars represent the 95% CI; *p < 0.05.

## Data Availability

All data reported in this paper are published and available at the following repository: DOI 10.17605/OSF.IO/CQPK7.
