# Supplementary material for: Superficial Ventral Premotor Pathways to Primary Motor Cortex Shape the Temporal Coordination of Precision Grasping
Source: Eur J Neurosci. 2026 Jul 2;64(1):e70597. doi: 10.1111/ejn.70597 (PMC13325708; doi:10.1111/ejn.70597)
Supplement: Supplementary file 1 — Figure S1: Main effect of “Time” on the AccP and DecP. The left panel shows the modulation in the AccP magnitude. The right panel shows the modulation of the DecP magnitude. After the PMv‐M1 cc‐PASAP we observe the reduction of the AccP and DecP magnitude. Error bars represent the 95% CI; *p < 0.05. Figure S2: “Time” main effect in the AccT. The main effect of time on the AT. After the PMv‐M1 cc‐PASAP we observe an increase in the AccT. Error bars represent the 95% CI; *p < 0.05. Figure S3: Modulation of the RT between the pre‐ and post‐30 sessions. Participants showed a significant reduction in RT in all conditions. As mentioned above, participants were instructed to perform a natural movement. This led to relatively long RTs but allows us to evaluate modulations without any experimental constraint. Error bars represent the 95% CI; *p < 0.05. Figure S4: Modulation of the MGA between pre‐and post‐30 session. The left panel shows a significant reduction in of the MGA in the “Informed” precision grip. The right panel shows the opposite trend in the power grip. Indeed, after the PMv‐M1 cc‐PASAP, there is a significant larger MGA. Here, the precision and power grip are plotted separately as the MGA strictly depend by the dimensions of the target. Collapsing the conditions into a single “Informed” condition would not be informative of the effects induced in the two actions. Error bars represent the 95% CI; *p < 0.05. [file EJN-64-0-s001.docx]

**Supplementary Material**

**Acceleration peak and Deceleration peak**

The ANOVA on AccP showed a significant main effect of “Action” (F_1,17_ = 10.28; p = 0.005; $\eta_{p}^{2}$ = 0.38) and “Time” (F_1,17_ = 6.29; p = 0.02; $\eta_{p}^{2}$ = 0.27; pre-PAS, M = 0.48 mm/s^2^, SD = 0.16; post-30, M = 0.43 mm/s^2^, SD = 0.14; **Figure S1**). This latter highlights a no-action specific significant reduction of AccP magnitude after the PMv-M1 cc-PAS_AP_. No significant main effect of “Information” emerged from the analyses (F_1,17_ = 1.16; p = 0.30; $\eta_{p}^{2}$ = 0.06). The interaction between “Action” and “Information” (F_1,17_ = 5.18; p = 0.04; $\eta_{p}^{2}$ = 0.23) was significant. Differently, the interaction between “Information” and “Time (F_1,17_ = 0.03; p = 0.88; $\eta_{p}^{2}$ = 0.002), “Action” and “Time” (F_1,17_ = 1.33; p = 0.27; $\eta_{p}^{2}$ = 0.07), and between the three factors (F_1,17_ = 0.23; p = 0.64; $\eta_{p}^{2}$ = 0.01) were non-significant.

The ANOVA on DecP showed a significant main effect of “Action” (F_1,17_ = 16.59; p = 0.001; $\eta_{p}^{2}$ = 0.49) and “Time” (F_1,17_ = 5.87; p = 0.03; $\eta_{p}^{2}$ = 0.26; pre-PAS, M = - 0.44 mm/s^2^, SD = 0.16; post-30, M = - 0.40 mm/s^2^, SD = 0.13). As for the AccP, this latter result showed a significant reduction of the DecP magnitude, after the PMv-M1 cc-PAS_AP_, in both actions. No significant main effect of “Information” emerged (F_1,17_ = 0.13; p = 0.72; $\eta_{p}^{2}$ = 0.01). No significant interaction was found between “Information” and “Action” (F_1,17_ = 0.70; p = 0.42; $\eta_{p}^{2}$ = 0.04), “Information” and “Time” (F_1,17_ = 0.01; p = 0.93; $\eta_{p}^{2}$ = 0.001), “Action” and “Time” (F_1,17_ = 0.01; p = 0.91; $\eta_{p}^{2}$ < 0.001) or between the three factors (F_1,17_ = 0.62; p = 0.44; $\eta_{p}^{2}$ = 0.04; **Figure S1**).

**
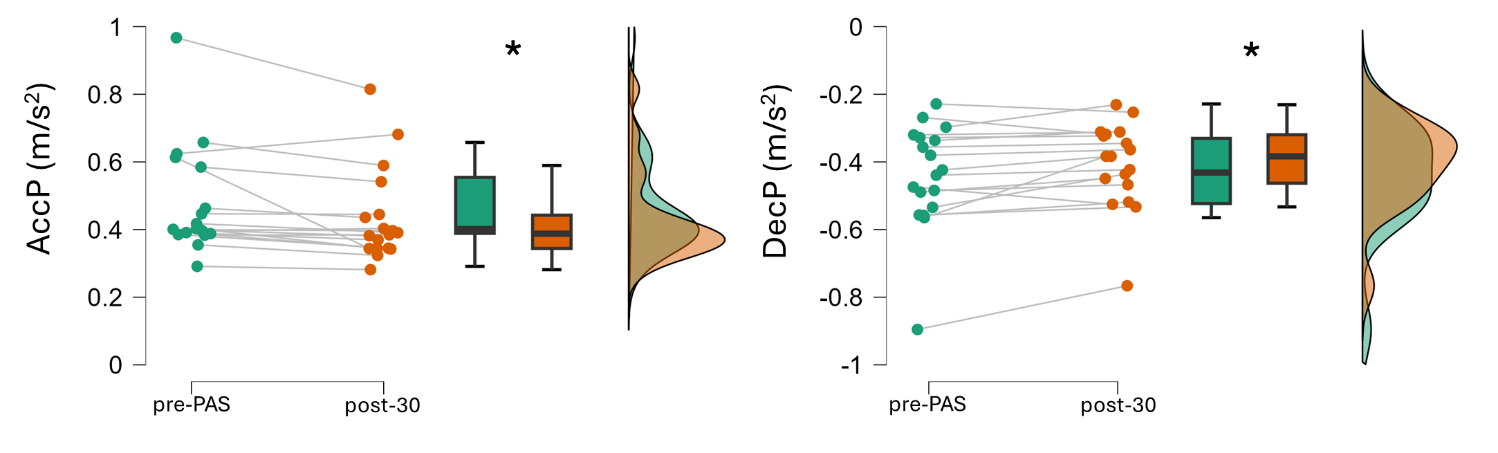
**

**Figure S1. Main effect of “Time” on the AccP and DecP**

The *left* panel shows the modulation in the AccP magnitude. The *right* panel shows the modulation of the DecP magnitude. After the PMv-M1 cc-PAS_AP_ we observe the reduction of the AccP and DecP magnitude. Error bars represent the 95% CI; * p < 0.05.

**Acceleration Time and Deceleration Time**

The ANOVA computed on the AccT data highlighted a significant main effect of “Time” (F_1,17_ = 7.20; p = 0.02; $\eta_{p}^{2}$ = 0.30; pre-PAS, M = 0.35 s, SD = 0.06; post-30, M = 0.37 s, SD = 0.07; **Figure S2**), “Information” (F_1,17_ = 33.57; p < 0.0001; $\eta_{p}^{2}$ = 0.66) and “Action” (F_1,17_ = 5.09; p = 0.04; $\eta_{p}^{2}$ = 0.23). No significant interaction was present between “Information” and “Action” (F_1,17_ = 2.01; p = 0.17; $\eta_{p}^{2}$ = 0.11), “Information” and “Time” (F_1,17_ = 0.91; p = 0.35; $\eta_{p}^{2}$ = 0.05), “Action” and “Time” (F_1,17_ = 0.19; p = 0.67; $\eta_{p}^{2}$ = 0.01) or between the three factors (F_1,17_ = 0.05; p = 0.82; $\eta_{p}^{2}$ = 0.003).

The ANOVA conducted on the DecT data showed a significant main effect of the “Action” factor (F_1,17_ = 11.73; p = 0.003; $\eta_{p}^{2}$ = 0.40) and no significant main effect of “Information” (F_1,17_ = 0.13; p = 0.73; $\eta_{p}^{2}$ = 0.01) and “Time” (F_1,17_ < 0.001; p = 0.99; $\eta_{p}^{2}$ < 0.001). No significant interaction was shown between “Information” and “Action” (F_1,17_ = 0.01; p = 0.92; $\eta_{p}^{2}$ = 0.001), “Information” and “Time” (F_1,17_ = 0.59; p = 0.45; $\eta_{p}^{2}$ = 0.03), “Action” and “Time” (F_1,17_ = 4.19; p = 0.06; $\eta_{p}^{2}$ = 0.20) or between the three factors (F_1,17_ = 1.00; p = 0.33; $\eta_{p}^{2}$ = 0.06).


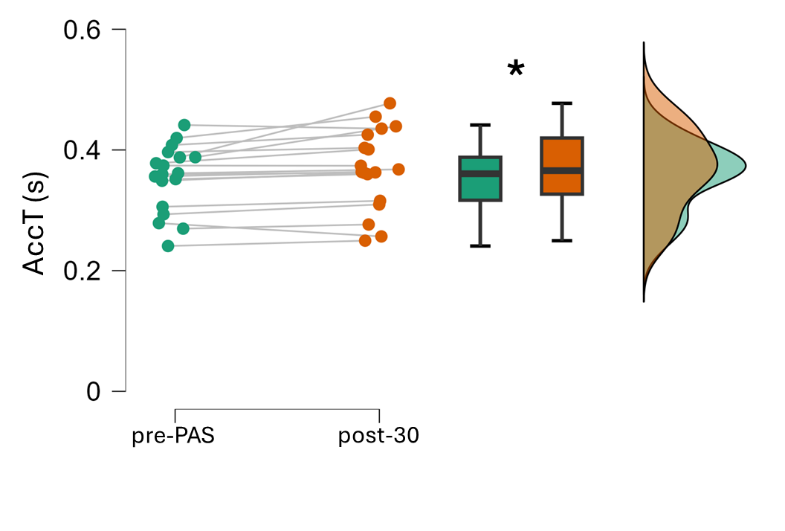


**Figure S2. “Time” main effect in the AccT**

The main effect of Time on the AT. After the PMv-M1 cc-PAS_AP_ we observe an increase in the AccT. Error bars represent the 95% CI; * p < 0.05.

**Peak Velocity, Average Velocity and Time to the peak velocity**

The ANOVA on the PV data showed no significant main effect of “Action” (F_1,17_ = 0.002; p = 0.97; $\eta_{p}^{2}$ < 0.01), “Information” (F_1,17_ = 0.84; p = 0.37; $\eta_{p}^{2}$ = 0.05) or “Time” (F_1,17_ = 2.08; p = 0.17; $\eta_{p}^{2}$ = 0.11), and no significant interaction between “Action” and “Information” (F_1,17_ = 0.81; p = 0.38; $\eta_{p}^{2}$ = 0.05), “Action” and “Time” (F_1,17_ = 0.004; p = 0.95; $\eta_{p}^{2}$ < 0.001), “Information” and “Time” (F_1,17_ = 0.08; p = 0.78; $\eta_{p}^{2}$ = 0.01) or between the three factors (F_1,17_ = 0.08; p = 0.78; $\eta_{p}^{2}$ = 0.01).

The ANOVA on the AV showed a significant main effect of “Action” (F_1,17_ = 4.94; p = 0.04; $\eta_{p}^{2}$ = 0.23) and “Information” (F_1,17_ = 5.15; p = 0.04; $\eta_{p}^{2}$ = 0.23), but no significant main effect of “Time” (F_1,17_ = 1.69; p = 0.21; $\eta_{p}^{2}$ = 0.09). No significant interaction emerged between “Action” and “Information” (F_1,17_ = 0.45; p = 0.51; $\eta_{p}^{2}$ = 0.03), “Action” and “Time” (F_1,17_ = 0.87; p = 0.37; $\eta_{p}^{2}$ = 0.05), “Information” and “Time” (F_1,17_ = 0.17; p = 0.69; $\eta_{p}^{2}$ = 0.01) or between the three factors (F_1,17_ = 0.02; p = 0.90; $\eta_{p}^{2}$ = 0.001).

The ANOVA on the TPV showed a significant main effect of “Information” (F_1,17_ = 6.63; p = 0.02; $\eta_{p}^{2}$ = 0.28) but a non-significant main effect of “Action” (F_1,17_ = 1.28; p = 0.27; $\eta_{p}^{2}$ = 0.07) or “Time” (F_1,17_ = 1.60; p = 0.22; $\eta_{p}^{2}$ = 0.09) factors. Similarly, no significant interaction emerged between “Action” and “Information” (F_1,17_ = 0.78; p = 0.39; $\eta_{p}^{2}$ = 0.04), “Action” and “Time” (F_1,17_ = 1.61; p = 0.22; $\eta_{p}^{2}$ = 0.09), “Information” and “Time” (F_1,17_ = 0.14; p = 0.71; $\eta_{p}^{2}$ = 0.01) or between the three factors (F_1,17_ = 0.13; p = 0.73; $\eta_{p}^{2}$ = 0.01).

**Movement Time and Reaction Time**

The ANOVA on the MT showed a significant main effect of “Action” (F_1,17_ = 24.99; p < 0.001; $\eta_{p}^{2}$ = 0.60) and “Information” (F_1,17_ = 16.43; p = 0.001; $\eta_{p}^{2}$ = 0.49), and no significant main effect of “Time” (F_1,17_ = 0.82; p = 0.38; $\eta_{p}^{2}$ = 0.05). Similarly, no significant interaction emerged between “Action” and “Information” (F_1,17_ = 0.13; p = 0.72; $\eta_{p}^{2}$ = 0.008), “Action” and “Time” (F_1,17_ = 2.12; p = 0.16; $\eta_{p}^{2}$ = 0.11), “Information” and “Time” (F_1,17_ = 0.10; p = 0.76; $\eta_{p}^{2}$ = 0.01) or between the three factors (F_1,17_ = 0.39; p = 0.54; $\eta_{p}^{2}$ = 0.02).

The ANOVA on the RT showed a significant main of “Time” (F_1,17_ = 7.90; p = 0.01; $\eta_{p}^{2}$ = 0.32; pre-PAS; M = 1.25 s, SD = 0.05; post-30, M = 1.22, SD = 0.05; **Figure S3**) and “Information” (F_1,17_ = 165.11; p < 0.001; $\eta_{p}^{2}$ = 0.91), but no significant main effect of “Action” (F_1,17_ = 0.18; p = 0.68; $\eta_{p}^{2}$ = 0.01). However, no significant interaction emerged between “Action” and “Information” (F_1,17_ = 0.44; p = 0.52; $\eta_{p}^{2}$ = 0.03), “Action” and “Time” (F_1,17_ = 2.39; p = 0.14; $\eta_{p}^{2}$ = 0.12), “Information” and “Time” (F_1,17_ = 1.41; p = 0.25; $\eta_{p}^{2}$ = 0.08) or between the three factors (F_1,17_ = 1.73; p = 0.21; $\eta_{p}^{2}$ = 0.09).


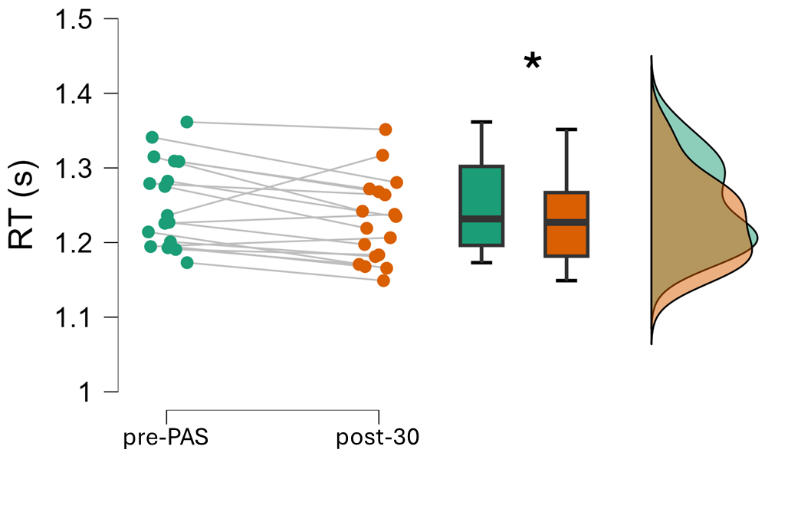


**Figure S3. Modulation of the RT between the pre- and post-30 sessions**

Participants showed a significant reduction in RT in all conditions. As mentioned above, participants were instructed to perform a natural movement. This led to relatively long RTs but allows us to evaluate modulations without any experimental constraint. Error bars represent the 95% CI; * p < 0.05.

**Maximum Grip Aperture**

The ANOVA on the MGA showed a significant main effect of “Action” (F_1,17_ = 259.47; p < 0.001; *η*^2^ = 0.94) and no significant main effect of “Information” (F_1,17_ = 3.03; p = 0.10; $\eta_{p}^{2}$ = 0.15) and “Time” (F_1,17_ = 0.10; p = 0.75; $\eta_{p}^{2}$ = 0.01). Moreover, a significant interaction between “Information" and “Time” emerged (F_1,17_ = 4.89; p = 0.04; $\eta_{p}^{2}$ = 0.22; **Figure S4**). No significant interaction emerged between “Action” and “Information” (F_1,17_ = 3.49; p = 0.08; $\eta_{p}^{2}$ = 0.17), “Action” and “Time” (F_1,17_ = 1.56; p = 0.23; $\eta_{p}^{2}$ = 0.08) or between the three factors (F_1,17_ = 0.07; p = 0.80; $\eta_{p}^{2}$ = 0.004). Considering the interaction between “Information" and “Time”, the Bonferroni-corrected post hoc analysis showed a significant modulation between the pre-PAS and post-30 session for the “Informed” (t_17_ = 2.46; p = 0.04) conditions and a non-significant modulation for the “Not-Informed” (t_17_ = -0.01; p > 0.99) conditions.


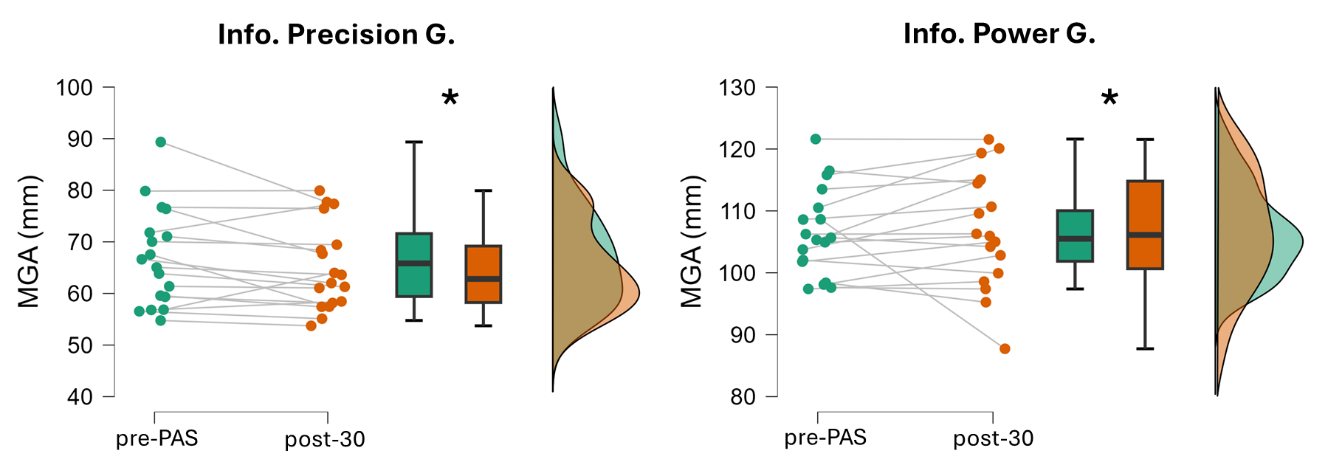


**Figure S4. Modulation of the MGA between pre-and post-30 session**

The *left* panel shows a significant reduction in of the MGA in the “Informed” precision grip. The *right* panel shows the opposite trend in the power grip. Indeed, after the PMv-M1 cc-PAS_AP_, there is a significant larger MGA. Here, the precision and power grip are plotted separately as the MGA strictly depend by the dimensions of the target. Collapsing the conditions into a single “Informed” condition would not be informative of the effects induced in the two actions. Error bars represent the 95% CI; * p < 0.05.
